# Screening of genes encoding adhesion factors and biofilm production in methicillin resistant strains of *Staphylococcus aureus* isolated from Palestinian patients

**DOI:** 10.1186/s12864-019-5929-1

**Published:** 2019-07-12

**Authors:** Kifaya Azmi, Walaa Qrei, Ziad Abdeen

**Affiliations:** 1 Biochemistry and Molecular Biology Department, Al-Quds Nutrition and Health Research Institute, Faculty of Medicine, Al-Quds University, Abu Deis, The West Bank Palestine; 2 Al-Quds Public Health Society, Jerusalem, Palestine; 3 Faculty of Medicine, Al-Quds Nutrition and Health Research Institute, Al-Quds University, Abu-Deis, P.O. Box 20760, The West Bank Palestine

**Keywords:** MRSA, Antibiotics, SCCmec, Biofilm, *Agr*, *sarA*

## Abstract

**Background:**

Intercellular adhesion and biofilm production by *Staphylococcus aureus* makes these bacteria resistant to antimicrobial therapy. Here, Methicillin-resistant *Staphylococcus aureus* (MRSA) strains were characterized and the prevalence of genes encoding adhesion factors and biofilm formation was determined.

**Results:**

All 248 MRSA isolates identified by cefoxitin disc diffusion were positive for the *mec*A gene. SCC*mec*-IV was the most frequently detected genotype (92.7%) and SCC*mec*-IVa was also very prevalent (84.3%). The quantitative microtiter plate assay showed that all the isolates were able to produce biofilm with levels ranging from high (21%) to moderate (46.4%) to low (32.7%).

All the strains possessed the *icaD/icaA genes* and produced biofilm (*P* < 0.05). None of the isolates possessed the *bap* gene*.* Furthermore, 94.8% of the isolates were positive for eno, 80.2% for *clfA* and for *clfB*, 78.2% for *fnbA*, 76.2% for *ebps*, 62.2% for *fib*, 39.9% for *cna and* 29.0% for *fnbB.* Also, nearly 69.8% of the isolates were positive for the gene *sarA*. All four *agr* groups were present: *agr* group 1 was predominant with 39.5%; agr group 3. *agr* group 2 and 3 strains carried more toxin-producing genes, and frequently produced more toxin. Sixty-six (26.6%) of the strains were multidrug resistant. All were vancomycin sensitive. *Agr* group I is more resistant to ciprofloxacin and gentamicin while *agr* group III is more resistant to erythromycin. Maximum sensitivity was to gentamicin and SXT, and they could be considered drugs of choice for controlling MRSA mediated infections in this region.

**Conclusions:**

Biofilm development in MRSA might be an *ica* dependent and one needs to investigate the involvement of other global regulators, *agr* and *sarA*, and their contribution to the biofilm phenotype, as the high rate of biofilm production among the studied strains of *S. aureus*.

## Background

Methicillin-resistant *Staphylococcus aureus* (MRSA) is a serious risk to hospitalized patients worldwide and characterized by its resistance to antimicrobial treatment, and more recently to vancomycin, the drug of last resort for many strains of MRSA. In addition to its bacterial antibiotic resistance, its ability to produce biofilm, a dynamic structurally complex multilayered cellular matrix is another important complicating factor. Understanding the molecular pathogenesis of *S. aureus* could assist in developing novel prevention and treatment strategies. Biofilm synthesis is necessary for the survival and persistence of MRSA in its hosts and is considered to be a major virulence factor [1] and one of many, including extracellular toxins and surface structures that are effective in the induction and continuance of infection in the host [2]. Biofilm production is important during infection, providing defense against several opposing mechanisms of host and protects the microorganisms from antimicrobial agents [3]. The ability to form biofilm is a trait associated with bacterial virulence and many chronic bacterial infections [4]. Several genes are involved in the manufacture and maintenance of biofilms by staphylococci, of which the most extensively studied are the *icaA* and *icaD* (intercellular adhesion A and B) genes responsible for the synthesis of polysaccharide intercellular adhesion (PIA) that includes N-acetylglucosamine as a main component of the exopolysaccharide matrix surrounding the bacterial cells within the biofilm [5–7]. The protein components of the microbial surface recognizing adhesive matrix molecules have a high ability to interact with the host extracellular matrix proteins like collagen-binding protein (*cna*), fibrinogen binding protein (*fib*), elastin binding protein (*ebpS*), laminin binding protein (*eno*), fibronectin binding proteins A and B (*fnbA* and *fnbB*) and clumping factors A and B (*clfA* and *clfB*) [8].

Several virulence determinants of *S. aureus* are under the control of two genetic loci namely *sarA* (staphylococcal accessory regulator) and the *agr* quorum-sensing system. *SarA* might impact methicillin-resistant *Staphylococcus aureus* (MRSA) persistence in such infections by up regulating the expression of many virulence factors including biofilm formation to facilitate evasion of the host immune system in late phases of growth. Inhibiting the production of *sarA* protein might influence the down regulation of biofilm and virulence factors [9].

Suppression of the *agr* quorum-sensing system is required for biofilm formation. Its recurrence in established biofilms either through addition of auto-inducing peptides (AIPs) or glucose depletion triggers biofilm detachment [10–12]. Bacteria of *S. aureus* fall into four polymorphic *agr* types (*agr* I, *agr* II, *agr* III, and *agr* IV) based on the specificity of the auto- inducing peptides (AIPs) with regard to the signal receptor *agr* C.

There are no data on either the virulence factors of the microbial surface components recognizing adhesive matrix molecules (MSCRAMM) family or factors responsible for biofilm formation in methicillin resistance *S. aureus* in Palestine. This study focused on exposing the genes encoding adhesion factors and biofilm-forming capacity, and those governing antibiotic resistances in strains of MRSA isolated from Palestinian patients. It also evaluated the correlation between biofilm production and the presence of *icaD*, SarA and agr genes in the clinical isolates.

## Results

### Characterization of MRSA strains and antibiotic susceptibility

From 2015 to 2018, 248 strains of MRSA: 78 (31.5%) from infected wounds; 34 (13.7%) from blood culture; 25 (10.1%) from nasal secretions; 23 (9.3%) from urine; 88 of various other origins were collected from major hospitals in the West Bank-Palestine.

By the cefoxitin disc-diffusion Resistance test (≤22 mm), the 248 bacterial isolates were identified as being phenotypically MRSA and confirmed them as such by targeting the *femA* and *mecA* genes, which, respectively, separate susceptible *S. aureus* from MRSA. All of the isolates tested positive for the *mecA* gene by a PCR assay.

The susceptibility patterns of methicillin resistant isolates to the other antimicrobials are presented in Table 1. The cefoxitin disc-diffusion test showed that all 248 isolates were resistant to methicillin and none were resistant to vancomycin. However, sensitivity was high to varying degrees to SXT, gentamicin, clindamycin, ciprofloxacin, and erythromycin that were, respectively, 77.8, 76.6, 61.7, 55.6 and 34.3% (Table 1).

**Table 1:** Frequency of antibiotic resistance of MRSA strains and biofilm

Antibiotics	Weak biofilm producer (0.07 < ODs ≤ 0.14)		Moderate biofilm producer (0.14 < ODs ≤ 0.28)		Strong biofilm producer (0.28 < ODs)		Total (*N* = 248)	
	S n. (%)	R n. (%)	S n. (%)	R n. (%)	S n. (%)	R n. (%)	S n. (%)	R n. (%)
Cefoxitin	0 (0%)	81 (32.7)	0 (0)	115 (46.3)	0 (0)	52 (20.9)	0 (0)	248 (100)
Penicillin	0 (0%)	81 (32.6)	0 (0)	115 (46.3)	0 (0)	52 (20.9)	0 (0)	248 (100)
Ciprofloxacin	49 (19.8%)	32 (12.9)	69 (27.8)	45 (18.1)	19 (7.6)	32 (12.9)	137 (55.2)	109 (43.9)
Clindamycin	50 (20.2%)	31 (12.5)	75 (30.2)	40 (16.1)	28 (11.2)	24 (9.7)	153 (61.6)	95 (38.3)
Erythromycin	29 (11.7%)	52 (20.9)	36 (14.5)	79 (31.8)	20 (8.0)	32 (12.9)	85 (34.2)	163 (65.7)
Gentamycin	69 (27.8%)	12 (4.8)	93 (37.5)	22 (8.8)	28 (11.2)	24 (9.6)	190 (76.6)	58 (23.3)
SXT	65 (26.2%)	16 (6.4)	92 (37.1)	23 (9.2)	36 (14.5)	16 (6.4)	193 (77.8)	55 (22.1)
Vancomycin	81 (32.7%)	0 (0)	115 (46.3)	0 (0)	52 (20.9)	0 (0)	248 (100)	0 (0)

Aside from cefoxitin and B-lactams antibiotics (penicillin G, amoxicillin/clavulanic acid, ceftriaxone and meropenem), the highest antibiotic resistance rates among the isolates of MRSA were for erythromycin (65.7%), ciprofloxacin (44.4%) and clindamycin (38.3%), followed by gentamycin (23.4%) and SXT (22.2%). All the isolates were susceptible to vancomycin (100%). Only, 66 (26.6%) isolates were MDR and, of 52 strongly positive biofilm-producing isolates, 20 (38.5%) were MDR and 32 (61.5%) were non-MDR.

Three different *SCCmec* types were detected among the isolates of MRSA that could be typed. Most of them carried SCC*mec* type IV (230/248, 92.7%), followed by SCCmec type I (11/248, 4.4%) and SCC*mec* type V (1/248, 0.4%)*.* Also, 2.4% of the isolates could not be typed by multiplex PCR. None of the isolates carried SCCmec type II or III. The majority of the strains carrying SCC*mec* type IV carried SCCmec-type IVa (84.3%), followed by type IVc (4.8%), type IVd (1.2%) and type IVb (0.4%), and three patients provided two strains of type IVa/IVc (1.2%). Isolates were classified as CA-MRSA when they possessed SCC*mec* IV, as one of the subtypes IVa, IVb, IVc, IVd, or SCCmec type V and 231 CA-MRSA strains were found among all the isolates. Out of 12 MRSA strains with SCCmec IVc, 9 and 7 isolates were resistant to erythromycin and clindamycin with weak biofilm producers. All but one of the SCCmec IVc were sensitive to gentamycin, ciprofloxacin and SXT.

### PCR screening of biofilm associated genes

Of the 248 strains of MRSA studied, 207 (83.5%) possessed the *ica*D gene and in 41 (16.5%) it was undetected. This percentage of *ica*D-negative strains was surprisingly high so detection of the *ica*A gene was undertaken which showed that all 41 *ica*D-negative strains were *ica*A-positive. High prevalence of *ica*A *and ica*D genes has shown a relationship to phenotypic biofilm formation.

None of the strains possessed the *bap* gene*.* The prevalence of *sarA*, *eno*, *clfA, clfB, fnbA*, *ebps*, *fib*, *cna*, and *fnbB* genes were 69.8, 94.8, 80.2, 80.2, 78.2, 76.2 62.2, 39.9 and 29.0%, respectively (Table 2). The frequency of *clfA*/*B* and *fnbB* genes of the *agr* group I were high at 92.9 and 52.0%, respectively. The frequency of *eno*, *fnbA*, *epbS*, *fib* and *cna* genes of the *agr* group III were also high at 97.6, 91.5, 84.1, 80.5 and 53.7%, respectively (Table 2).

**Table 2:** The presence of biofilm-related genes for each *agr* group

	Agr group (%)					
Biofilm Plate Titer (N = 248)	AgrI	AgrII	AgrIII	AgrIV	NT	SarA gene
Weak biofilm producer (*n* = 81, 32.7%)	30.6	17.4	43.9	66.7	13.9	77.7
Modertae biofilm producer (*n* = 115, 46.3%)	42.9	56.5	50.0	22.2	47.2	73.9
Strong biofilm producer (*n* = 52, 20.9%)	26.5	26.1	6.1	11.1	38.9	48.1
Total (n, %)	(98, 39.5%)	(23, 9.3%)	(82, 33.1%)	(9, 3.6%)	(36, 14.5%)	173
*SarA gene* (173, 69.7%)	80.6	65.2	80.5	77.7	16.6	
Adhesion Genes						
icaD/ icaA(n = 248)	87.7	87.0	78.0	88.9	80.6	58.5
Eno (*n* = 235, 94.8%)	92.9	91.3	97.6	100.0	94.4	71.5
ClfA (*n* = 199, 80.2%)	92.9	87.0	89.0	55.6	27.8	80.4
ClfB (n = 199, 80.2%)	92.9	87.0	89.0	55.6	27.8	80.4
FnbA (*n* = 194, 78.2%)	87.8	78.3	91.5	66.7	25.0	81.4
EbpS (*n* = 191, 76.2%)	82.7	60.9	84.1	55.6	55.6	76.4
Fib (*n* = 154, 62.2%)	60.2	73.9	80.5	66.7	16.7	83.1
Cna (*n* = 99, 39.9%)	46.9	21.7	53.7	0.0	11.1	78.8
FnbB (*n* = 72, 29.0%)	52.0	21.7	13.4	11.1	11.1	81.9
Antibiotic Resistance						
Cefoxitin/Penicillin (n = 248)	100.0	100.0	100.0	100.0	100.0	100
Erythromycin (*n* = 163)	59.2	60.9	69.5	33.3	86.1	63.2
Ciprofloxacin (*n* = 109)	58.2	56.5	20.7	33.3	52.8	63.3
Clindamycin (*n* = 95)	28.6	43.5	48.8	11.1	44.4	65.3
Gentamycin (*n* = 58)	27.6	21.7	11.0	22.2	41.7	55.2
SXT (*n* = 55)	19.4	21.7	11.0	11.1	58.3	41.8
MDR (*n* = 66)	22.4	43.5	13.4	11.1	61.1	46.9
Source of isolate						
Wound (*n* = 78)	36.7	34.8	35.4	22.2	8.3	80.7
Blood (*n* = 34)	11.2	17.4	11.0	11.1	25.0	52.9
Nasal (*n* = 25)	11.2	13.0	8.5	0.0	11.1	64
Pus (n = 23)	8.2	8.7	13.4	22.2	0.0	95.6
Urine (n = 23)	1.0	0.0	11.0	22.2	30.6	43.5
Tissue (*n* = 13)	9.2	8.7	2.4	0.0	0.0	61.5
Sputum (*n* = 12)	3.1	8.7	6.1	0.0	5.6	58.3
Other sites (*n* = 40)	19.4	8.7	12.2	22.2	19.4	72.5

The coexistence of the studied virulence genes was investigated in the 248 clinical isolates of MRSA, only nine of which had all the genes investigated. Five had only the *eno* gene. Despite these low prevalence rates, the other 234 strains possessed at least one other virulence gene.

Of the clinical isolates in which most virulence genes were investigated for coexistence, 56 were positive for all the genes but one, either *eno* (*n* = 29) or *fnbB* (*n* = 27) gene. Of 42 clinical isolates, 18 strains were negative for two genes, *fnbB* and *fib* genes and 24 were negative for *finbB* and *cna* genes.

### Determination of biofilm production by the microtiter plate method

All the strains of MRSA produced biofilm. In the microtiter plate method for determining this and using the mean OD570 of the negative control (0.07), values between 0.07 and 0.140 (2 × the negative control value of 0.07), were considered to be strains that were weak producers, which accounted for 81 (32.7%) strains, values between 0.140 and here 0.280 (4 × the negative control value of 0.07) to be moderate producers, which accounted for 115 (46.4%) strains and values higher than 0.280 were strong producers, which accounted for 52 (21.0%) strains (Table 3).

**Table 3:** Biofilm-forming capacity of 248 methicillin-resistant strains of *Staphylococcus aureus* (MRSA) and percentages of their adhesion genes related to antibiotics

Biofilm	Adhesion gene	Gentamycin %	Ciprofloaxin %	SXT %	Erythromycin %	Clindamycin %	MDR %	Total %
Strong (n = 52, 20.9%)	FinbB	23.1	23.1	1.9	15.4	11.5	7.7	46.2
	ClfA/ClfB	32.7	40.4	11.5	36.5	28.8	19.2	69.2
	Ebps	42.3	46.2	21.2	44.2	34.6	32.7	71.2
	Can	15.4	19.2	3.8	15.4	11.5	7.7	38.5
	Eno	46.2	59.6	30.8	57.7	25.0	38.5	94.2
	Icad	42.3	57.7	26.9	51.9	36.5	32.7	88.5
	FinbA	34.6	44.2	13.5	38.5	26.9	21.2	71.2
	Fib	26.9	34.6	5.8	28.8	21.2	17.3	46.2
Moderate (n = 115, 46.3%)	FinbB	5.2	18.3	0.9	17.4	6.1	6.1	26.1
	ClfA/ClfB	13.0	30.4	13.0	54.8	28.7	17.4	81.7
	Ebps	12.2	31.3	13.9	53.0	27.0	18.3	79.1
	Can	7.0	7.8	8.7	28.7	17.4	8.7	40.9
	Eno	18.3	36.5	19.1	65.2	33.0	26.1	94.8
	Icad	13.9	32.2	17.4	56.5	29.6	21.7	82.6
	FinbA	9.6	28.7	9.6	50.4	24.3	12.2	78.3
	Fib	7.8	26.1	7.0	43.5	22.6	10.4	64.3
Weak (n = 81, 32.6%)	FinbB	3.7	14.8	7.4	24.7	8.6	6.2	88.9
	ClfA/ClfB	13.6	34.6	66.7	51.9	33.3	17.3	82.7
	CLFB	13.6	34.6	17.3	54.3	33.3	17.3	86.4
	Ebps	11.1	30.9	14.8	46.9	30.9	14.8	75.3
	Can	7.4	11.1	7.4	23.5	17.3	7.4	39.5
	Eno	12.3	37.0	17.3	59.3	37.0	17.3	95.1
	Icad	12.3	30.9	14.8	51.9	33.3	18.5	81.5
	FinbA	13.6	30.9	14.8	51.9	33.3	17.3	82.7
	Fib	9.9	29.6	8.6	45.7	28.4	11.1	69.1

Among the 65.7 and 43.9% of high resistance of the strains of MRSA to erythromycin and ciprofloxacin, 31.8 and 18.1% were moderate biofilm producers., respectively.

**Fig. 1:**
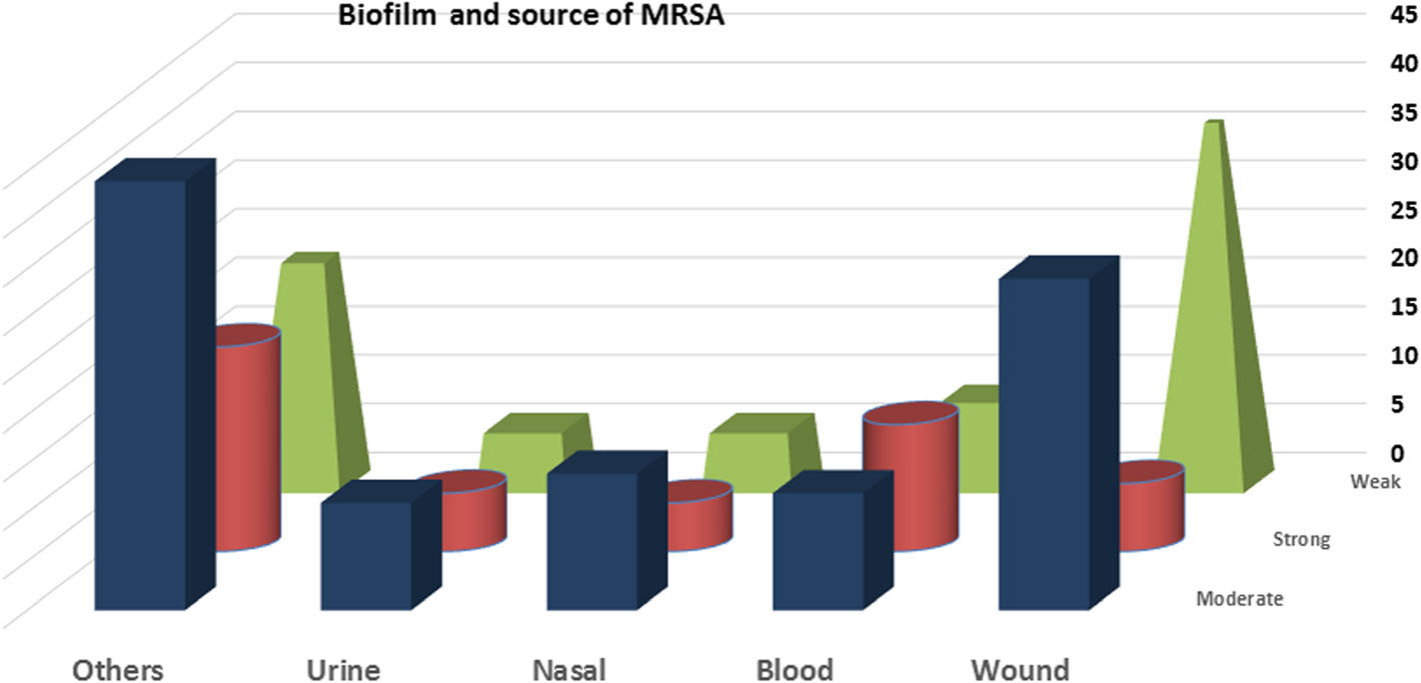
Biofilm phenotype and the source of strains of MRSA, i. e. tissues and lesions

Among the 78 clinical isolates of *S. aureus* from wounds, 37 (47.4%) were weak producers of biofilm, 34 (43.6%) were moderate producers and 7 (9%) were strong producers. Of the 34 isolates from blood, 9 (26.5%) were weak producers, 12 (35.3%) were moderate producers and 13 (38.2%) were strong producers. Of the 23 isolates from urine, 6 (26.1%) were weak producers, eleven (5.5%) were moderate producers, and 6 (26.1%) were strong producers. Of the 25 nasal isolates, 6 (24%) were weak producers, 14 (56%) were moderate producers and 5 (20%) were strong producers. Among the 88 isolates obtained from other different clinical samples, 23 (26.1%) were weak producers, 44 (50%) were moderate producers and 21 (23.9%) were strong producers, of which half of the sputum samples were strong producers (Fig. 1).

All the virulence genes were found in weak, moderate and strong producers of biofilm. The least frequent genes were the *cna* (39.9%) and *fnbB* (29%) genes, the percentages of which were, respectively, 39.5 and 29.6% for weak biofilm producers, 40.8 and 20.1% for moderate biofilm producers, and 38.4 and 34.6% for strong biofilm producers (Table 3).

### The *agr* groups

Table 2 gives the *agr* groups of the strains of MRSA. The 248 strains separated into four *agr* groups with 98 (39.5%) belonging to *agr*-I, making it the predominant type, 23 (9.3%) belonging to *agr*-II, 82 (33.1%) belonging to *agr*-II, 9 (3.6%) belonging to *agr*-IV and 36 (14.5%) were negative regarding the *agr* PCR. There was no relationship between *agr* specific groups and the genes encoding MSCRAMM. Strains that belonged to the *agr*-I group showed higher antibiotic resistance to ciprofloxacin and gentamycin, compared to the other three *agr* groups. Strains that belonged to *agr*-III group had higher erythromycin (69.5%) and clindamycin (48.8%) resistance compared to the other *agr* groups. Of the 66 MDR strains, 20 belonged to the *agr*-I group and only 10, 11 and one belonged to the *agr-*II, *agr-*III and *agr-*IV groups, respectively (Table 2).

### Detection of the *SarA* gene

The *sarA* gene was found in 173 (69.8%) strains of MRSA. Regarding phenotypic biofilm formation, 63 of 81 (77.7%) were weak producers of biofilm, 85 of 115 (73.9%) strains were moderate producers and 25 of 52 (48.1%) were strong producers with a significant difference (*P* < 0.05). There was a high prevalence of *fib* (83.4%), *clfB* and *clfB* (80.4% each) and *fnbB* (81.9%) and *fnbA* (81.4%). Of the strains of MRSA carrying the *sarA* gene, 46.9% were MDR, and 63.2, 63.3 and 65.3%, 55.2 and 41.8% were resistant to erythromycin, ciprofloxacin, clindamycin, gentamycin and SXT., respectively (Tables 2, 3).

## Discussion

Biofilm production by *S. aureus* has been identified as the most important means of defense against host antagonistic responses. Beside enabling bacterial colonization of host tissues, it also prevents clearance of the bacteria by antimicrobial agents and host immune responses [13], leading to morbidity and mortality owing to the metastatic spread of abscesses [14]. Here the ability among strains of MRSA isolated from hospitalized- and out-patients to form biofilm was studied, combining it with their clinical molecular biological details and determining the presence of genes encoding these virulence factors and its relation to antibiotics. The SCC*mec* type IV was the most frequent SCC*mec* type among the strains. Its presence in the sporadic strains among the 92.7% and the group of strains from out-patients shows their great persistence [15]. SCC*mec* type IV is currently one of the most frequent nosocomial SCC*mec* types and is found in several countries [16, 17].

The antimicrobial resistance patterns of strains of this type varied considerably.

Here, 26.6% of the strains of MRSA with multi-resistance to more than three antibiotics were of the SCC*mec* type IV with 83.4% carried SCC*mec* type IVa and all are biofilm producers. These results indicate that the production of biofilm might be one of the crucial factors increasing resistance to commonly used antibiotics. That deserve a special comment. This higher MDR rely to protective nature of the biofilm, the bacteria growing in it are internally resistant to many antibiotics and the antibiotic resistance in the strains of the bacteria residing in biofilm could increase up to 1000 times as seen by Neupane and colleagues [18]. The main reasons for this may be difficulty in penetration of biofilm by antibiotics, slow growth rate of the bacteria and presence of antibiotic degradation mechanisms.

Moreover, the high resistance of the strains of MRSA to erythromycin, ciprofloxacin,of which were moderate biofilm producers with higher rate of studied adhesion genes, particularly, eno gene. This agreed with the high prevalence of drug resistance presented in a study done in Iran where the resistance of strains of MRSA to ciprofloxacin, erythromycin and gentamicin was 51.28, 87.18 and 71.8%, respectively [19]. It seems that misuse and overuse of some antibiotics, including gentamicin, clindamycin, ciprofloxacin and erythromycin, have caused a high prevalence of resistance to them in this region, showing that empirical treatment of infections of strains of MRSA at Palestinian hospitals with these antibiotics may not be effective and they should not be used and considered first-line drugs for the treatment of infections of MRSA in the local population. Appropriate measures are needed to prevent treatment failures. All the strains were susceptible to vancomycin and more than two third of the strains were susceptible to trimethoprim sulfamethoxazole. Vancomycin is reported to be the most effective antibiotic for Gram-positive bacteria, including MRSA, but reduced susceptibility to both antibiotics has been reported in some studies [20, 21]. Vancomycin and other glycopeptides have remained the last options for eradication of infections caused by *S. aureus*. The data presented here also showed all the strains, producing biofilm, were sensitive to vancomycin. This is consistent with other researchers’ recommendation that vancomycin, which is a very expensive drug, is the last antibiotic option and should be used sparingly.

The data presented here agree with susceptibility rates in other countries [22]. Wang and colleagues [23] reported a 78.6% susceptibility rate to trimethoprim-sulfamethoxazole among strains of MRSA, which is of concern and emphasizes the need for persistent monitoring of the development of antimicrobial resistance by strains of *S. aureus* that leads to community- and hospital-acquired infections. Here, we report a high rate of SXT resistance (22.1%), which in the future could increase as a consequence of horizontal transmissibility of the dfrK gene, encoding for trimethoprim-resistance.

Here, phenotypic and genotypic evaluations, PCR and crystal violet (CV) staining assays, were combined to detect biofilm production in strains of *S. aureus*.. All the strains were biofilm producers with variation in biofilm mass. To understand the molecular mechanism of biofilm production by strains of MRSA, in addition to the *icaD*/*icaA*, *sarA* and *agr* group genes, the frequency of nine selected genes involved in biofilm production were detected. Many studies have shown the role and requirement of the intracellular adhesion locus (ica) in biofilm production [24, 25]. The *icaA* and *icaD* genes determine the ability of strains of *S. aureus* to produce biofilm by mediating the synthesis of PIA which suggest that the ica locus would be a good target in the therapy of implant infections. There was a 100% agreement between the genotypes and phenotypes of strains where all the strains having *icaD*/*icaA* and producing biofilm, which agreed with the findings of Liberto and colleagues [26] and support those of Namvar and colleagues [27], who reported that strains of *S. aureus* had no ability to produce biofilm, unless they were positive for the *icaD* gene. Similar observations were reported by Grinholc and colleagues [28], who found that 91% of strains of MRSA possessed the *icaD* gene. Contrarily, Arciola and colleagues [29], detected *icaA* and *icaD* genes in only 61% of strains. The relatively low percentage of *icaD* positive strains described by Arciola and colleagues [29] resulted from the method of detection they used, in which primers complementary to the sequence of the *icaD* gene from *Staphylococcus epidermidis*, rather than primers complementary to the sequences of the *icaD* and *icaA* genes from *S. aureus* were used. There was no difference in the distribution of the *ica* genes in strongly and weakly virulent strains, which agreed with the findings of others [5, 30, 31]. The PIA mediates intercellular adherence and accumulation of multilayer biofilms. In our study the ica operon was present in all MRSA strains but strains differed in biofilm mass. It is suggested that these strains also used other systems to form biofilm such as protein A (SpA) or fibronectin binding proteins.

Other contradictory published data stated that some strains, in spite of the presence of the *ica* locus, do not produce biofilm [25]. Recently, it has become evident that the presence of PIA is not essential for the production of biofilm in many strains of MRSA [32].

The ability to produce biofilm varied among the strains of MRSA and also greatly among the other different genotypes of *S. aureus* where an increasing number of different adhesion molecules have been found. The frequency of *eno, clfA/clfB, fnbA, ebps, fib, cna, and fnbB* genes was found to be 94.8, 80.2, 78.2, 76.2, 62.2, 39.9 and 29.0%, respectively (Table 2). While in other studies [33], showed the frequency of *eno, clfA/clfB, fnbA, ebps, fib, cna and fnbB* genes in strains of MRSA was 79, 97, 64, 12, 76, 56, and 51%., respectively. Yang and colleagues [34], showed the prevalence of genes associated with biofilm in the ST59-SCCmec IV-t437 clone were *icaA* (100.0%), *icaD* (97.3%), *fnbpA* (100.0%)*, fnbpB* (0), *clfA* (100%), *clfB* (100%), *cna (*2.7%), *bbp* (0), *ebpS* (88.5%). This explains the discrepancies between studies, which is related to differences in the frequency of clones among different countries. The gene *bap*, whose protein was, probably, the first protein shown to have a role in biofilm production in *S. aureus*, was not tracked in our study. It is said to be absent in all strains, which agrees with the study by Serray and colleagues [35]. However, the absence of *bap* indicates that the *ica*-dependent mechanism, might be primarily responsible for adhesion and biofilm production in strains as suggested by Vautor and colleagues [36].

The *fnbA* and *fnbB* genes appear to be essential to the invasion and adhesion of bacteria and might be correlated with their biofilm-producing ability. In this study, a low percentage (29.0%) of occurrence of the *fnbB* gene was observed.

However, Arciola and colleagues [29], found a high occurrence of this gene (99.5%). This could partly be ascribed to the different region of the locus analyzed by the couple of primers*.* However, the *fnbA* gene was detected in 76.2% of the strains, which is similar to what was observed by Ikawaty and colleagues [28]. There is a significant difference between strains from blood and those from wounds regarding the presence of the *fnbB* gene. About a third of the strains (35.9%) from wounds carried the gene. However, only 14.7% of the strains from blood carried the *fnbB* gene. A comparative analysis between strains of MRSA and strains of MSSA showed that the *fnbpA* gene was more likely to be present in strains of MRSA whereas the *fnbB* gene was more likely to be present in strains of MSSA [34]. However, other studies did not find a correlation between methicillin resistance and the prevalence of genes associated with biofilm [37]. This disagreement may be owing to the specific clonal complexes of strains that might contain an exclusive combination of surface-associated and regulatory genes [38].

This study indicated that the *clfA* and the *clfB* genes were present in 82.7% of the strains and constituted the bound coagulase of *S. aureus*. This study showed that the strains from all sources except urine (43.5%) had a high percentage of, both, the *clfA* and the *clfB* genes.

Elastin is the main component of elastic fibers, which are proteins that provide strength and flexibility to connective tissue and is highly expressed in the lung, skin and blood vessels and widely expressed at low levels in most mammalian tissues [39]. Elastin binding protein of *S. aureus* (EbpS), said to facilitate binding of bacteria to elastin rich host, extracellular cell matrix (ECM) [40]. *EbpS* is a cell-surface molecule the mediates binding of a bacterial cell to soluble elastin peptides and tropoelastin [39]. The presence of the *ebpS* gene was found in 76.2% of the strains studied here. Another gene, shown to have a crucial role in binding to extracellular matrix, fibrinogen (fib), was also detected in 62.2% of the strains. This agreed with the findings of Pereyra and colleagues [41] who reported higher percentages of 90 and 71.7%, respectively. This contradicted the findings of [35], where the *fib* and *ebpS* genes were detected at a rates of 5.66 and 9.34% of strains. The difference in the prevalence of these genes is probably owing to the distribution of variants of the genotype of *S. aureus* in different countries. The incidence of *c*na was 39.9% in the strains of MRSA studied here. This agreed with the findings of Nashev and colleagues in Italy [36], and in Bulgaria [29], who reported of similar rates of 46.7% and occurrence of this gene (11.32%) was reported by serray and colleagues [35].

The expression of several virulence factors of *S. aureus* was shown to be controlled by certain genetic loci, particularly, the staphylococcal accessory regulator, which consists of the *sarA* gene and accessory gene regulator (*agr*) locus (Jarraud et al., 2002). In studies done by other colleagues [42–44] all their strains of MRSA harboring the *icaADBC* genes were positive for the *sarA* gene, which was contradicted by this study.

The *ica* genes are regulated by multiple genes such as the *sarA*, and *agr* genes. They might interact with each other and regulate biofilm production. The *sarA* gene has an effect on many virulence genes of *S. aureus* and appears to be a master controller of biofilm production, promoting synthesis of fibronectin and fibrinogen binding proteins and also toxins for tissue spread while repressing expression of protein A and the four major extracellular proteases governed by the *SspA*, *SspB*, *Au*r, and *ScpA* genes [2, 45]. Here, about two third of the strains genotypically possessed the *sarA* gene and phenotypically produced biofilm and the *fnbB* gene was common among strains that were strong producers of biofilm (34.6%) suggesting the importance of PIA-independent biofilm production in these strains. Interestingly in this study, a higher rate of the strains of MRSA possessed the *fnbA*, *fnbB* and the *fib* gene which were also positive for the *sarA* gene.

Different levels of *sarA* expression in clinical isolates of *S. aureus* have been related to differences in extracellular protease production [46] and that *sarA* can directly and positively regulate levels of *fnbA* transcription [47]. On the other hand, Pozzi and colleagues [48] reported that biofilm production in strains of MSSA mainly occurs via PIA synthesis while in strains of MRSA it is related more to adhesion owing to the *fnbB* gene. So further investigation and studies are needed.

Strains producing biofilm have a very high tendency to exhibit antimicrobial multidrug resistance. However, 46.9% of the strains with the *sarA* gene were MDR, and more than halfwere shown to be resistant to erythromycin, ciprofloxacin, clindamycin and gentamycin This makes *sarA* an attractive target for antimicrobial drug development [49, 50]. Surprisingly, most of the isolates from wounds and pus were *sarA* positive.

To date, strains of *S. aureus* have been classified into four main groups, *agr* -I to *agr*-IV, according to differences in their *agr* genes, (Jarraud et al., 2002). The central role of the *agr*-encoded quorum-sensing system in the regulation of virulence makes it an attractive target for antimicrobial drug development. However, mutations in the *agr* gene or interference with *agr* gene activity by a cross-inhibiting *agr* pheromone can promote the production of colonization factors like MSCRAMMs and biofilm development [6]. All four *agr* groups were found among the strains studied here, with *agr* group I in large proportion and more than half of the samples from wounds belonged to this group.

Previous studies also found the *agr* group I to be the predominant type [51]. Here, 14.5%strains could not be typed by the same method, possibly, owing to a deletion in the *agr* locus. It is noteworthy that the strains in *agr* group III had a greater number of the *fnbA*, *ebps*, *cna*, *eno* and *fib* genes, and most of the toxin producing strains also belonged to *agr* group III while the strains in *agr* group-I had a greater number of *fnbB*, *clfA* and c*lfB*. Regarding the relationship between *agr* group-III and biofilm production, the data revealed that strains belonging to *agr* group-III had a greater number of weak and moderate producers of biofilm compared to those belonging to *agr* group-I, which, interestingly, had more and stronger biofilm-producing strains. Also, the strains belonging to *agr* group-III had higher antibiotic resistance to erythromycin and clindamycin compared to those belonging to *agr* group-I, which carried a greater number of strains resistant to ciprofloxacin (58.2%), gentamycin (27.6%) and SXT (19.4%). The presence of the combination of genes studied here, where 3.9% of the strains possessed all the genes examined and including the *icaA* and *icaD* genes, could mean that they might have a selective advantage, *e*. g. a good genetic capacity for adherence and better colonization of hosts. Moreover, the coexistence of *icaA*, *icaD, agr and sarA* and eight MSCRAMM genes in 11.7%of the strains agree with the findings of Tristan and colleagues [52]. The most common biofilm gene combination among the strains of MRSA was that of *agr*, *sarA*, *eno*, *clfA*/c*lfB, fnbA*, *ebps* and *fib* genes. The mechanism of multidrug resistance is said to result from close cell to cell contact in the biofilm that makes the transfer of plasmids containing MDR genes among them easier, which limits the therapeutic options, creating an economic and social burden to the healthcare system. Biofilm development is a very complicated process that involves numerous factors. The present survey study is a first step. It provides preliminary results for further detailed future studies. One limitation of the study, is the inability to use control *S. aureus* strains that lack each of the gene tested in this study. Mutants defective in either; IcaA, IcaD, agr, saR, each of the genes that code for the MSCRAAM proteins. This way, biofilm development by the mutant would be measured directly to that produced by the tested isolates.

## Conclusion

The present study revealed that MRSA strains isolated from clinical materials from hospitalized patients produced biofilm and possessed *icaA* and *icaD* genes, with differed biofilm mass, indicating that these strains may also use other system to form biofilm. The high rate of biofilm production among the strains of *S. aureus* and high rate of drug resistance among the biofilm producing strains, detection of biofilm adhesion genes indicating staphylococcal virulence markers and showing that the burden of MRSA in the Palestinian West Bank region was high. Further, clinical strains of *S. aureus* and the ability of several strains of MRSA to produce biofilm in the absence of *sarA* and *agr* genes needs further investigation to clarify the mechanism underlying the production of biofilm independent of the activity of the *sarA* and *agr* genes. On the basis of the antimicrobial susceptibility testing, aworrisome increase in erythrocyte and ciprofloxacin resistance was observed, which deserves future attention.

## Methods

### Clinical strains

A Total of 248 strains of MRSA were isolated from patients admitted to four Palestinian hospitals located in, Jerusalem, Ramallah, Bethlehem and Nablus. The study period was between November 2015 and April 2018. Most came from the Al-Makassed Islamic Charitable Society Hospital in Jerusalem. MRSA ATCC 4300 and *S. epidermidis* ATCC 12228 were reference strains provided by Dr. Adham abu Taha of the Palestinian Al Najah University. All the strains were stored at − 80 °C in brain-heart infusion (BHI) (Himedia, Mumbai, India) plus 25% glycerol (EMPROVE, Darmstadt, Germany). This study was approved by the Research Ethical Committee of Al-Quds University. Written and informed consents were sent for the participating hospitals and clinics.

### Identification of isolates

All isolates were identified by classic microbiological methods: colony morphology; mannitol fermentation; Gram staining, catalase test; coagulase test. Antibiotic susceptibility was determined by the disc-diffusion method (Oxoid, Basingstoke, UK).

The antibiotics used in this study were cefoxitin (30 μg), penicillin (10 U), amoxicillin/clavulanic acid (10 μg), ceftriaxone (30 μg), meropenem (10 μg), erythromycin (15 μg), ciprofloxacin (5 μg), clindamycin (10 μg), gentamicin (10 μg), SXT (25 μg) and vancomycin (30 μg). Apart from β-lactam, multi drug resistance (MDR) for MRSA was defined as resistance to at least three of the antimicrobial agents.

Isolates were classified as susceptible or resistant to methicillin according to the criteria of the Performance Standards for Antimicrobial Susceptibility Testing (2002). Methicillin resistant strains of *S. aureus* were detected by the disk-diffusion method, using a cefoxitin (FOX) disk (30 μg) on Mueller-Hinton agar plates according to the Clinical Laboratory Standards Institute (CLSI) guidelines [53]. An infection was considered healthcare-associated if the date of the infection occurred on or after the third day of admission to an inpatient facility.

### Genomic DNA extraction

Genomic DNA was extracted from overnight fresh cultures on Trypticase Soy Broth (TSB), using either a ‘Nucleospin’ DNA extraction kit (Macherey-Nagel, Germany) [54] or a Presto Mini gDNA Bacteria Kit (Geneaid).

### Molecular typing

#### Detection of the *mecA* gene and SCC*mec* typing by PCR

The *mecA* gene and *femA* endogenous control gene were amplified in the same reaction. The primers used to amplify the *mecA* gene were *mec*A1F (5′-GTAGAAATGACTGAACGTCCGATAA-3′) and *mec*A2R (5′-CCAATTCCACATTGTTTCGGTCTAA-3′) [16]. The primers used to amplify the femA gene were femA GFEMAR-1(5′-AAAAAAGCACATAACAAGCG-‘3) and femA GFEMAR-2 (5’-GATAAAGAAGAAACCAGCAG-‘3) [55]. Each reaction used 1 μM of each primer and 2 μl of DNA, and was performed in Thermo Scientific Reddy Mix PCR mater Mix conc 2X in a final volume of 25 μl. The thermal cycling program for detecting both genes was: one cycle of initial denaturation at 95 °C for 15 min; 34 cycles of denaturation at 95 °C for 30 s; annealing at 58 °C for 30 s; extension at 72 °C for 1 min; a final extension at 72 °C for 5 min.

The amplified products (*femA*: 132 bp and *mecA*: 310 bp) were resolved in a 2.5% agarose gel. The fragments were stained with ethidium bromide and visualized and photographed using a gel documentation system. A 100 bp ladder was run as a molecular weight marker. Isolates that were confirmed to be methicillin sensitive by the disk diffusion method and then by the absence of the *mec*A gene were excluded from this study.

Exposing the existence of SCC*mec* types and subtypes I, II, III, IVa, IVb, IVc, IVd, and V of all the isolates of MRSA was done by the multiplex PCR assay described by Boye and colleagues [56], which used 9 pairs of primers that are unique and specific for the above mentioned SCC*mec* types and subtypes. Exposing the existence of SCCmec subtype IV was done by the multiple PCR assay described by Zhang et al. [57]. Isolates unable to be typed were designated NT. Amplification was performed as described by Hadyeh and colleagues (2019).

#### Detection of biofilm genes

Simplex and multiplex PCRs were used to detect the following genes in all the isolates of MRSA: *bap* (encoding biofilm-associated protein); *ebpS* (encoding elastin-binding protein); *eno* (encoding laminin-binding protein); *fib* (encoding fibrinogen-binding protein); *fnbA* (encoding fibronectin-binding protein A); *fnbB* (encoding fibronectin-binding protein B); *clfA and clfB* (encoding clumping factors A and B); *cna* (encoding collagen-binding protein)*.* The specific primers and PCR thermal profiles used for these genes were as described by others [3, 35, 52]. The amplified products *cna*: 423 bp; *ebpS*: 652 bp; *eno*: 302 bp; *fnbA*: 127 bp; *fnbB*: 524 bp; *fib*: 404 bp; *bap*: 971 bp; *clfA*: 292 bp and *clfB*: 205 bp were resolved in a 2.5% agarose gels.

#### Detection of icaD and icaA genes

The presence of *icaD* DNA was detected as described by Gowrishankar and colleagues [58]. The specific forward primer was *icaD* (5’ATG GTC AAG CCC AGA CAG AG3′) and the specific reverse primer was icaD (5’CGT GTT TTC AAC ATT TAA TGC AA3’). For *icaD*-negative strains, detection of the *icaA* gene was done using the forward primer *icaAF* (5’ACA CTT GCT GGC GCA GTC AA 3’) and reverse primer *icaAR* (5’TCTGGAACCAACATCCAACA3’) as proposed by [30]. The *icaD* and *icaA* genes were amplified by a PCR to generate 188 bp and 198 bp fragments, respectively.

### Determination of *agr* group and *sarA* gene

The *agr* typing was done by a multiplex-PCR to determine the *agr* allele types I to IV, using the *agr* group specific primers and amplification conditions as described by [30]. The *agr* system groups were classified based on the hyper-variable domain of the *agr* locus and their responding receptors separated into four major *agr* groups. *Pan-agr*, corresponding to the conserved sequences of *agrB*, was used in all the reactions.

Based on the *agr* locus polymorphism, four reverse primers were used, each specific for the amplification of a single *agr* group. The *agr* groups were identified by amplicon size: 440 bp for *agr* I; 572 bp for *agr* II; 406 bp for *agr* III; 588 bp for *agr* IV.

*SarA* DNA was detected, using the forward primer sarAF (5’CCCAGAAATACA ATCACTGTG’3) and reverse primer sarAR (5′ AGTGCCATTAGTGCAAAACC’3) as described by Gowrishankar and colleagues [58], which produced an amplicon of 720 bp.

### Biofilm formation assay

The isolates of MRSA were tested for biofilm formation. The assay was performed in polystyrene 96-well microtiter plates that had flat-bottomed wells that were stained with crystal violet according to Stepanovic and colleagues [59]. *Staphylococcus epidermidis* ATCC 12228, and MRSA ATCC 43300 were used as biofilm-producing controls. Trypticase soy broth medium was used as a negative control to determine background OD. The microtiter plate method was done as described by Atshan and colleagues [60]. The amount of biofilm formed was estimated by reading the optical density (OD) at 570 (630) nm and recording the absorbance using a microplate reader (RT-2100C, Rayto, IVD). The average OD value of each triplicate of experimental samples and negative controls was calculated. Biofilm formation was separated into four categories according to [19]: 1, ODs ≤ ODc = no biofilm produced, therefore a non-producer; 2, ODc ≤ ODs ≤ 2× ODc = weak biofilm produced, therefore a weak producer; 3, 2× ODc ≤ ODs ≤ 4 × ODc = moderate biofilm produced, therefore a moderate producer; 4, 4× ODc < ODs = strong biofilm produced, therefore a strong producer, where ODc = OD of the negative control and ODs = OD of the experimental samples.

### Statistical analysis

Data analysis was done using SPSS software version 20.0 (IBM, Armonk, USA). Pearson’s chi-square was used in the statistical analysis. A *P* value less than 0.05 was considered statistically significant.

## Abbreviations

BURP: Based upon Repeat Pattern
CA-MRSA: Community-associated Methicillin Resistant *Staphylococcus aureus*
*clfA* and *clfB*: Clumping factors A and B.
*ebpS*: Elastin binding protein
*eno*: Laminin binding protein
*fib*: Fibrinogen binding protein
*fnbA* and *fnbB*: Fibronectin binding proteins A and B
MDR: Multi-drug resistant
MRSA: Methicillin resistant *Staphylococcus aureus*
PCR: Polymerase chain reaction
PVL: Panton–Valentine leucocidin
*sarA*: Staphylococcal accessory regulator
*Cna*: Collagen-binding protein
SCC*mec*: Staphylococcal chromosome cassette *mec*
